# Threonine Phosphorylation of an Electrochemical Peptide-Based Sensor to Achieve Improved Uranyl Ion Binding Affinity

**DOI:** 10.3390/bios12110961

**Published:** 2022-11-02

**Authors:** Channing C. Thompson, Rebecca Y. Lai

**Affiliations:** Department of Chemistry, University of Nebraska-Lincoln, Lincoln, NE 68588-0304, USA

**Keywords:** calmodulin, electrochemical metal ion sensor, peptide design, phosphothreonine, self-assembled monolayer, uranyl ion

## Abstract

We have successfully designed a uranyl ion (U(VI)-specific peptide and used it in the fabrication of an electrochemical sensor. The 12-amino acid peptide sequence, (n) DKDGDGYIpTAAE (c), originates from calmodulin, a Ca(II)-binding protein, and contains a phosphothreonine that enhances the sequence’s affinity for U(VI) over Ca(II). The sensing mechanism of this U(VI) sensor is similar to other electrochemical peptide-based sensors, which relies on the change in the flexibility of the peptide probe upon interacting with the target. The sensor was systematically characterized using alternating current voltammetry (ACV) and cyclic voltammetry. Its limit of detection was 50 nM, which is lower than the United States Environmental Protection Agency maximum contaminant level for uranium. The signal saturation time was ~40 min. In addition, it showed minimal cross-reactivity when tested against nine different metal ions, including Ca(II), Mg(II), Pb(II), Hg(II), Cu(II), Fe(II), Zn(II), Cd(II), and Cr(VI). Its reusability and ability to function in diluted aquifer and drinking water samples were further confirmed and validated. The response of the sensor fabricated with the same peptide sequence but with a nonphosphorylated threonine was also analyzed, substantiating the positive effects of threonine phosphorylation on U(VI) binding. This study places emphasis on strategic utilization of non-standard amino acids in the design of metal ion-chelating peptides, which will further diversify the types of peptide recognition elements available for metal ion sensing applications.

## 1. Introduction

Uranium has become an emerging heavy metal contaminant because of its potential nephrotoxicity and osteotoxicity [1,2]. Although it is not one of the most abundant elements in the earth’s crust, traces of it can be found in rocks, soils, and various water sources. Redox transitions of uranium in sediments determine its mobility in the environment [3,4]. Human exposure to uranium is likely via consuming water from uranium-rich groundwater resources. Continuous exposure of uranium from any source can increase a person’s risk of kidney damage [1,2]. Overexposure to uranium also increases a person’s estimated lifetime risk of cancer. Among the different forms of uranium, hexavalent uranium (U(VI)) is the most mobile. Mobility of uranium has also been linked to the presence of nitrate, a groundwater contaminant that originates from chemical fertilizers and animal waste [5]. Other anthropogenic activities such as mining and milling have also contributed to the elevation of uranium concentration in environmental waters. Recent studies have highlighted the issues with uranium contamination in the United States and that there are sites where the uranium level is extremely high [5]. Therefore, it is necessary to regulate the amount of uranium allowed in potable water. To address this issue, the United States Environmental Protection Agency (EPA) has set a maximum contaminant level (MCL) of 30 µg/L (~126 nM) for uranium in public drinking water in 2003 [6]. However, the maximum contaminant level goal remains at zero, placing uranium in the same category as lead and arsenic.

Owing to the health concerns associated with uranium exposure, there is a critical need to monitor the level of uranium in public drinking water on a regular basis. To meet this demand, several analytical techniques have been developed for detecting and monitoring uranium, specifically UO_2_^2+^ (U(VI)). These techniques include inductively coupled plasma mass spectrometry [7,8], UV spectrophotometry [9,10,11], fluorescence spectroscopy [12], and surface enhanced Raman spectroscopy (SERS) [13,14]. Various electrochemical U(VI) detection approaches have also been reported recently [15,16,17,18]. Most electrochemical detection methods involve the use of modified electrodes. For example, Guo et al. employed a polydopamine/reduced graphene oxide nanocomposite-modified glassy carbon electrode for detection of U(VI) [17]. Biomaterials have also been used; Jarczewska et al. reported a DNA-based detection approach that exploits the interactions between U(VI) and the DNA phosphate backbone in 2014 [16]. A peptide-mediated detection method that uses α-hemolysin nanopores was developed later in 2017 [18]. While many of these approaches have achieved low limit of detections (LODs), they either require multistep detection processes or the addition of relatively complex nanomaterials and reagents. For real world sensing applications, it is ideal to employ detection probes that are already modified with a reporter molecule. Thus, despite the progress made towards the design and fabrication of U(VI) sensors, there is still a demand for new sensing platforms that are user-friendly, cost-effective, and compatible with complex sample matrices.

In the past years, a wide range of folding- and dynamics-based electrochemical sensors has been developed for metal ion detection. One of the studies focused on the use of an oligoadenine probe for Au(III) detection, whereas another study employed an oligothymine probe for the analysis of Hg(II) [19,20]. A cytosine-containing sequence was also reported to be capable of interacting with Ag(I) [21]. An electrochemical aptamer-based (E-AB) Cd(II) sensor that employed a 36-base DNA aptamer was later reported in 2017 [22]. These sensors possess high sensitivity, specificity, and can be used in a wide range of complex samples [23,24]. While there are many examples of DNA (E-DNA)-based metal ion sensors, there are fewer reports of peptide-based metal ion sensors in the literature [19,20,21,22,23,24,25,26,27,28]. Peptides are important biorecognition elements, and they have been employed for detection of many other targets, from volatile organic compounds to viral protein and clinically relevant proteases [29,30,31]. They have also been used to create antifouling surfaces for target detection in complex biological systems [32]. For the current sensing platform, the use of peptides as biorecognition elements could broaden the range of detectable target analytes and is thus a research area worth further exploration.

Here, we have designed a peptide probe with high affinity for U(VI) and used it to fabricate an electrochemical peptide-based (E-PB) sensor. The 12-amino acid (AA) probe sequence is derived from calmodulin (CaM), a Ca(II)-binding protein, and contains a phosphothreonine that enhances its affinity for U(VI) over Ca(II), its native target [33]. The use of short peptides instead of intact proteins in the fabrication of E-PB and other surface-based sensors has multiple advantages. First, because of their small size, they can form a self-assembled monolayer (SAM) with high probe density, thereby expanding the dynamic range of the sensor. Second, short peptides do not suffer from issues such as denaturation and improper immobilization orientation. Although interactions between U(VI) and various peptides have been investigated to elucidate their in vivo toxicity [34], few have been employed in sensor applications. This work describes a promising alternative to detecting U(VI), an important yet often overlooked environmental contaminant, in complex samples in a simple and cost-effective manner. It also highlights the benefits of exploring different binding motifs in metal ion-binding proteins, including the use of non-standard and non-protein AAs, in designing metal ion sensors [35].

## 2. Materials and Methods

### 2.1. Materials

Chemicals and metal ion standards were purchased from Sigma (Sigma-Aldrich, St. Louis, MO, USA) and used as received. The U(VI) (UO_2_(NO_3_)_2_) standard was purchased from Ricca Chemical Company (Arlington, TX, USA). The deionized (DI) water used in this study was purified by the Millipore Synergy Ultrapure Water System (Billerica, MA, USA). The sensor interrogation buffer, Phys2, contained 20 mM Tris-HCl, 140 mM sodium chloride, and 5 mM potassium chloride (pH 7.3). Several experiments were performed in 50% synthetic aquifer and drinking water samples. The synthetic aquifer sample contained specific ions found in the South Dakota Minnelusa aquifer [36,37]. The synthetic aquifer sample was subsequently diluted with a Phys2 buffer in a 1:1 ratio. The synthetic tap water sample contained 30 mg L^−1^ calcium, 0.02 mg L^−1^ iron, 9.0 mg L^−1^ magnesium, 4.9 mg L^−1^ potassium, 28 mg L^−1^ sodium, 0.05 mg L^−1^ zinc, 0.10 mg L^−1^ copper, and 0.14 mg L^−1^ phosphorous [38]. This sample was further diluted with a 2x Phys2 buffer in a 1:1 ratio prior to being used in the experiment.

The thiolated and methylene blue (MB)-modified peptide probes were purchased from Xaia Peptides (Göttenborg, Sweden). In brief, both peptides were synthesized using a fully automated multiple synthesizer (Syro II from MultiSyntech GmbH, Germany) with Fmoc (9-fluorenylmethyl-oxycarbonyl) methodology. A coupling agent, 2-(1H-Benzotriazole-1-yl)-1,1,3,3-tetramethyluro-nium hexafluorophosphate, was used. N-α-Fmoc-N-ε-1-(4,4-dimethyl-2,6-dioxocyclohex-1-ylidene)-3-methylbutyl-L-lysine (Fmoc-Lys(ivDde)-OH) was first coupled to an insoluble resin support, followed by the remaining AAs, and a 6-(Tritylthio)hexanoic acid was coupled to the last AA in the probe sequence. To cleave the ivDde group, the resin was mixed with 2% hydrazine in dimethylformamide. All other protecting groups were found to be stable against this treatment. In the last step, MB was coupled to the epsilon amino group of the lysine residue with 2-(1H-benzotriazol-1-yl)-1,1,3,3-tetramethyluronium hexafluorophosphate as the coupling agent.

The synthesized peptides were subsequently cleaved and deprotected from the solid support by treatment with 90% trifluoroacetic acid (TFA), 8% tri-isopropylsilane, and 2% water (*v*/*v*/*v*) for 2.5 h at room temperature. The products were then precipitated in ether. The crude materials were purified by preparative HPLC on a Kromasil 100-10-C18 reverse phase column (30 × 250 mm) using an eluent of 0.1% TFA in water (A) and 80% acetonitrile in water (B). The peptides were eluted with a successive linear gradient of 10% B to 80% B in 30 min at a flow rate of 23 mL min^−1^. The fractions corresponding to the purified peptides were lyophilized. This one-step purification by reverse-phase HPLC was sufficient to obtain peptides with >95% purity. Both peptides were shipped and stored in the lyophilized state. They were later reconstituted in DI water before use.

The structures and sequences of the two probes are shown below and also in Figure S1 (in Supplementary Materials):

U-pT-12: (n) HS-(CH_2_)_6_-DKDGDGYI**pT**AAE-K-MB (c)

U-12: (n) HS-(CH_2_)_6_-DKDGDGYI**T**AAE-K-MB (c)

* phosphothreonine and threonine are bold and underlined

### 2.2. Fabrication of U-pT-12 and U-12 Sensors on Gold Disk Electrodes

To fabricate the sensors, 2-mm diameter gold disk electrodes purchased from CH Instruments, Inc. (Austin, TX, USA) were polished and cleaned according to a previously established procedure [27]. In brief, the electrode was polished with 0.1 μm diamond slurry (Buehler, Lake Bluff, IL, USA), rinsed with DI water, and sonicated for 5 min to remove the adhered particulates. Next, it was electrochemically cleaned by redox cycling in 0.5 M sulfuric acid (H_2_SO_4_). The exact area of the electrode was calculated using the charge from the gold oxide reduction peak obtained in cyclic voltammetry (CV) in 0.05 M H_2_SO_4_. The roughness factor (exact area/geometric area) of the electrodes was between 1.0 and 1.2.

To fabricate the U-pT-12 sensor, a clean electrode was placed in an ethanol solution containing 10 µM U-pT-12 for 1 h at 4 °C. Next, the electrode was rinsed with DI water, gently dried with N_2_, and placed in 2 mM C6-OH for 17–19 h at 4 °C. A nonphosphorylated peptide probe (U-12) was also used in the fabrication of a U(VI) sensor. To fabricate the U-12 sensor, a clean electrode was first placed in an ethanol solution with 1 µM U-12 for 1 h at 4 °C. Next, the electrode was rinsed with DI water, gently dried with N_2_, and placed in 2 mM C6-OH for 17–19 h at 4 °C. The modified electrodes/sensors were then placed in an electrochemical cell with 2 mL of Phys2 buffer. These sensors were stable for ~10 h, and they could be reproducibly fabricated using these two fabrication procedures (e.g., 90% similarity in probe coverage).

### 2.3. Fabrication of a U-pT-12 Sensor on a Paper Electrode

A previously established method was used to deposit gold on a 3-mm diameter screen-printed carbon electrode (SPCE) [27,39]. The gold-modified electrode was electrochemically cleaned, and the roughness factor was estimated to be ~7. To fabricate the sensor, an ethanol solution with 10 µM U-pT-12 was drop-casted onto the electrode for 1 h. The partially modified electrode was subsequently placed in an ethanol solution containing 2 mM C6-OH for 17–19 h. 

### 2.4. Sensor Characterization and Target Interrogation

All electrochemical measurements were carried out at room temperature (~22 °C) using our standard experimental setup [27,40,41]. The working electrode was either a 2-mm diameter polycrystalline gold disk electrode or a 3-mm diameter SPCE (CH Instruments, Inc., Austin, TX). A conventional Ag/AgCl (3 M KCl) electrode and a platinum wire were used as the reference and counter electrodes, respectively. Both alternating current voltammetry (ACV) and CV were used to characterize the sensors [40,41]. The sensors were first interrogated with 4 µM U(VI) in a Phys2 buffer. Sensor regeneration was accomplished by placing the electrode in 0.5 M HCl for 4 min. The concentrations of U(VI) used in the calibration experiment were 50, 100, 250, 500, 750, 1000, 1750, 2500, and 4500 nM. An equilibration time of 47 min was used for all nine data points. The LOD is the lowest target concentration capable of generating a signal change that is significantly different (i.e., s/n = 3) from the signal fluctuation in the absence of the target [27]. In addition to U(VI), the U-pT-12 sensor was tested against nine other metal ions, Ca(II), Mg(II), Pb(II), Hg(II), Cu(II), Fe(II), Zn(II), Cd(II), and Cr(VI), each at a concentration of 4 µM. Higher concentrations of Ca(II), the native target of CaM, were also used in a separate experiment [33]. The probe coverage and percent signal suppression (%SS) were calculated using equations reported in our previous studies [27,40,41]. Other than the ACVs and CVs, all data presented in this study are averages from three separate experiments.

## 3. Results and Discussion

### 3.1. Sensor Design 

**Figure 1:**
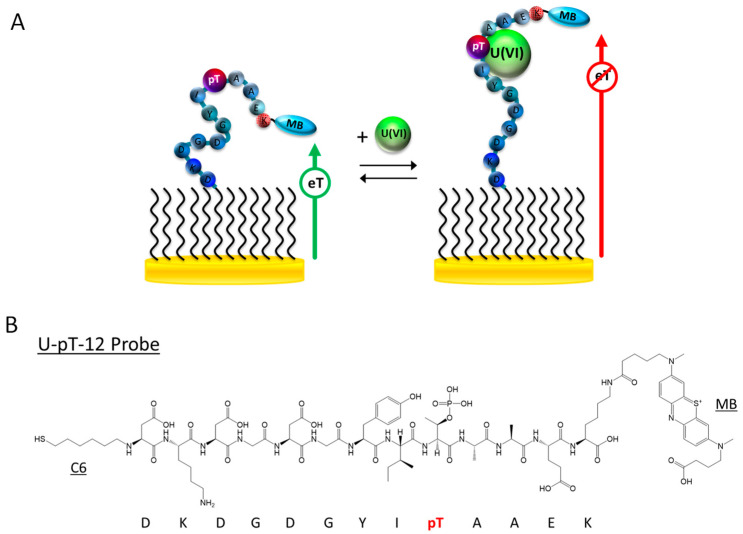
(**A**) Schematic representation and sensing mechanism of the E-PB U(VI) sensor. (**B**) Structure of the thiolated and MB-modified U-pT-12 peptide probe.

The U-pT-12 peptide sequence is derived from CaM, a ubiquitous and highly conserved Ca(II)-binding protein responsible for regulating a wide array of enzymes [42]. It possesses two pairs of EF-hand motifs in two domains that are separated by an α-helix [43]. The Ca(II) recognition sites in CaM have been characterized using different techniques, such as X-ray crystallography and NMR [44,45]. Although Ca(II) is the main target, CaM has also been characterized for its interactions with other metal ions, including Cd(II) and Pb(II) [46]. More recently, Pardoux et al. studied the U(VI) binding properties of the metal binding loop of the EF-hand motif of CaM [33]. Specifically, they assessed the effects of threonine phosphorylation on U(VI) binding and showed that the EF-hand motif with a phosphothreonine at position 9 of the loop has significantly higher affinity (~100×) for U(VI). The peptide forms a 1:1 complex with U(VI) and with a dissociation constant (*K*_d_) of 0.25 ± 0.06 nM at pH 7 [33]. Furthermore, it has a much higher affinity for U(VI) than Ca(II), its native target. Because of the aforementioned properties, this 12-AA peptide sequence can potentially be used as a biorecognition probe for detection of U(VI) (Figure 1B and Figure S1A).

To fabricate the E-PB U(VI) sensor, we first immobilized the dual-modified U-pT-12 probe on a gold electrode surface. Like other E-PB sensors, the detection mechanism is based on the change in peptide flexibility upon interacting with the target [27,47,48,49,50]. The peptide probe is relatively flexible prior to interacting with U(VI), and the current from the tethered MB label is high. In the presence of U(VI), the peptide probe rigidifies, producing a large attenuation in the MB current (Figure 1). Unlike other E-B sensors we previously developed where the binding pocket cannot be accurately determined, the U(VI) binding pocket is known for this peptide. According to the previously reported Fourier transform infrared spectroscopy results, the deprotonated phosphoryl group of the phosphothreonine side chain is directly involved in uranyl coordination [33]. The binding pocket is thus located at or near the phosphothreonine residue (9th residue in the peptide probe) (Figure 1B and Figure S1A). U-12, a dual-labeled peptide probe with a nonphosphorylated threonine, was also designed to provide a comparison with the U-pT-12 probe (Figures S1B and S2A).

### 3.2. Sensor Characterization

**Figure 2:**
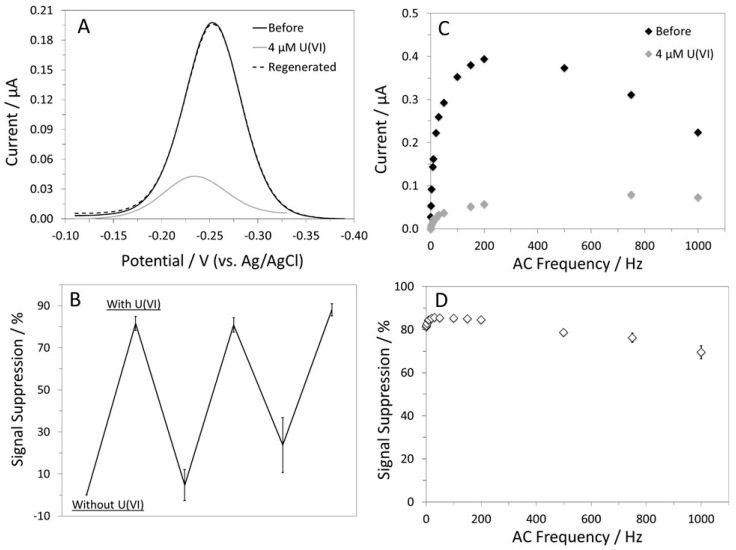
(**A**) ACVs of the U-pT-12 sensor recorded at 10 Hz in a Phys2 buffer. (**B**) Sensor reusability plot. (**C**) AC frequency-dependent responses of the sensor and (**D**) %SS plot. The error bars are not visible because of the size of the data label.

ACV was first used to characterize the U-pT-12 sensor, and the resultant voltammograms are shown in Figure 2A [47,48,49,50]. The sensor responded well to U(VI), showing 78% SS in the MB peak current. This large signal attenuation alludes to favorable interactions between U(VI) and the peptide. These interactions rigidifies the peptide structure, restricting the access of the redox label to the electrode surface. In addition to the change in the MB peak current, there was a noticeable shift in the peak potential, suggesting a change in the solution pH [51,52]. This shift is presumably due to the addition of the U(VI) standard solution, which was made in 3% nitric acid. In this case, the initial pH of the buffer was pH 7.3, but it dropped to 7.0 upon the addition of 4 µM U(VI). The addition of an equal amount of 3% nitric acid without U(VI) also resulted in a similar change in the MB peak potential. In comparison to other electrochemical metal ion sensors, the response of the U-pT-12 sensor was slower; signal saturation was achieved in ~40 min [22,27]. This could be, in part, due to the size of the target as well as the probe coverage. For this sensor, the probe coverage well-suited for target binding was ~1.1×10^12^ molecules cm^−2^. Although sensors with a lower probe coverage responded faster to the target, they were not used in this study because of the low %SS and limited monolayer stability [40,41]. To provide a comparison, we tested the U-12 sensor under the same experiment condition (Figure S2). As shown in Figure S2B, the sensor did not respond optimally to U(VI), and the %SS was only 15%. These results highlight the importance of threonine phosphorylation in the recognition of U(VI) in the current sensor design.

The U-pT-12 sensor, the sensor that responded well to U(VI), was further tested for its ability to be regenerated and reused. It was successfully regenerated after being placed in 0.5 M HCl for 4 min (Figure 2A). Protonating the phosphoryl group of the phosphothreonine side chain is clearly effective in disrupting the interactions between the U(VI) and the peptide probe. After the first regeneration, the sensor was reused for two more times; however, the responses of the sensor in subsequent uses were slightly lower (Figure 2B). This change in %SS is likely due to the lack of complete removal of bound U(VI) and/or loss of surface-immobilized peptide probes. Although other sensor regeneration methods were attempted, 0.5 M HCl was considered the most effective regeneration reagent for this sensor.

The response of the U-pT-12 sensor is also dependent on the applied AC frequency [47,48,49,50,53,54]. In the absence of U(VI), the MB peak current increased rapidly from 1 to 200 Hz and decreased steadily at 200 Hz and beyond. In the presence of U(VI), the MB current plateaued at 750 Hz, but the change was more gradual, especially between 50 and 750 Hz. The reduction in the MB current was minimal above 750 Hz (Figure 2C). Overall, the change in the MB current was less dependent on the frequency in the target-bound state, which is consistent with the behavior of other “signal-off” sensors [40,41]. The change in %SS with frequency was also determined for the U-pT-12 sensor. As can be seen in Figure 2D, moderately high AC frequencies (e.g., 8–200 Hz) resulted in the highest %SS, but 10 Hz was considered the optimum frequency. Although a larger %SS can be achieved at higher frequencies, they were not used because of the sloping and noisy baseline.

**Figure 3:**
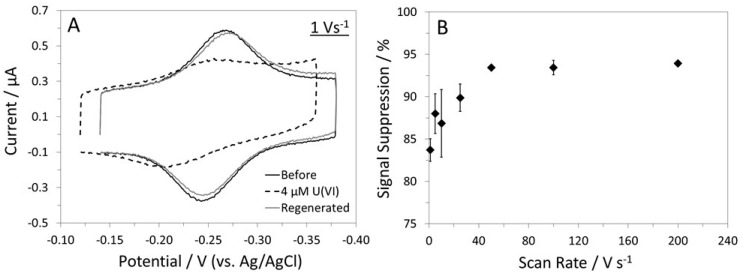
(**A**) CVs of the U-pT-12 sensor in a Phys2 buffer. (**B**) CV scan rate-dependent %SS plot. These results were obtained by analyzing the cathodic peak currents.

We also assessed the U-pT-12 sensor’s performance in CV (Figure 3). Overall, the sensor responded well to U(VI), as indicated by the large decrease in the MB signal. The MB peak current was found to be dependent on the scan rate (Figure S3). For example, prior to target addition, the cathodic peak current varied linearly with scan rate from 1 to 100 V s^−1^ but deviated from linearity above 100 V s^−1^. The trend remained relatively unaffected after the addition of U(VI) even though the current was significantly lower. A scan rate-dependent %SS plot was obtained by analyzing the MB peak currents before and after the addition of U(VI) (Figure 3B). Although a larger signal change was observed at scan rates beyond 50 V s^−1^, the MB peak current was very small when compared to the capacitive current at those scan rates. Thus, a lower scan rate such as 1 V s^−1^ was considered ideal for the U-pT-12 sensor. Both cathodic and anodic peak currents can be used to characterize the sensor, and the results were found to be similar. Overall, the CV results provide further support for the proposed “single-off” sensing mechanism.

### 3.3. Sensor Performance: Sensitivity, Specificity, and Selectivity

**Figure 4:**
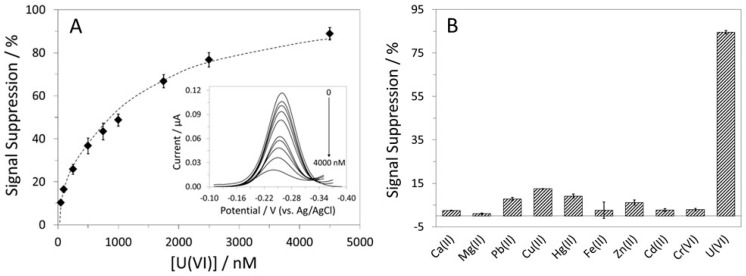
(**A**) Calibration curve for the U-pT-12 sensor and (inset) the resultant ACVs. (**B**) The responses of the sensor to nine metal ions and U(VI), each at a concentration of 4 µM.

Although both ACV and CV are suitable sensor interrogation techniques, we considered ACV to be superior to CV in this case because of the ease in determining the MB peak current. ACV was thus used to further characterize the U-pT-12 sensor. The calibration curve for the sensor is shown in Figure 4A, and the corresponding voltammograms are included in the inset. The LOD for U(VI) was 50 nM, and the dynamic range was from 50 to 4500 nM. A higher target concentration was not used because of the extreme shift in the MB peak potential, a consequence of the lowered solution pH (i.e., breaking the buffering ability). The calibration curve was fitted to a one-site binding model, and the *K*_d_ was estimated to be 971(±147) nM. This value is significantly higher than the value reported by Pardoux et al. [33], which is expected given that the peptide probe could behave rather differently once immobilized on a surface and with neighboring probes in close proximity. Although the LOD is higher than some of the reported values, it is still much lower than the MCL of 30 µg/L (~126 nM) for uranium in public drinking water. 

To determine the U-pT-12 sensor’s specificity for U(VI), we analyzed its responses to nine other metal ions: Ca(II), Mg(II), Pb(II), Cu(II), Hg(II), Zn(II), Fe(II), Cr(VI), and Cd(II) (Figure 4B). Among the tested metal ions, Pb(II), Cu(II), and Hg(II) were found to cross-react to a small extent, but the %SS were much lower than that obtained for U(VI). Importantly, it responded minimally to Ca(II), the native target of CaM. It is noteworthy that the response to Ca(II) was very limited even at relatively high concentrations. Furthermore, it responded well to U(VI) even in the presence of 4000 µM Ca(II), showing 76% SS (Figure S4). Owing to the incorporation of a phosphothreonine, this peptide probe clearly has a higher affinity for U(VI) when compared to Ca(II) [33]. 

**Figure 5:**
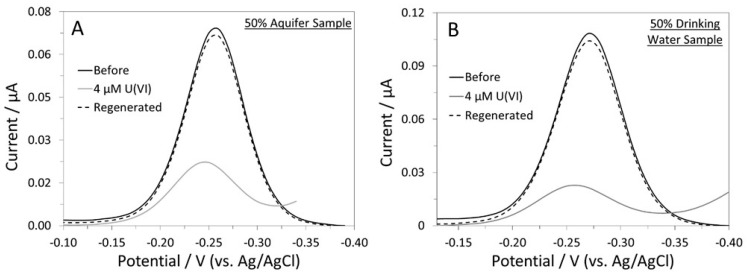
ACVs of the U-pT-12 sensor in (**A**) a 50% synthetic aquifer sample and (**B**) a 50% synthetic drinking water sample.

The sensor’s response to U(VI) in a 50% synthetic aquifer sample was also investigated [36]. As shown in Figure 5A, the sensor responded well to the spiked U(VI); the %SS was 75%, which corresponds to a target recovery rate of 96%. The disparities in pH and ionic strength between the pure buffer and the 50% aquifer sample could be the reason behind the reduced sensor response. The sensor showed close to complete signal regeneration after being placed in 0.5 M HCl for 4 min. To verify the potential use of this sensor for drinking water analysis, we tested it in a 50% synthetic drinking water sample [38]. The sensor responded well to U(VI) (77% SS), and the target recovery rate was estimated to be 99% (Figure 5B). It was also regenerated using the aforementioned method. Overall, despite the slightly lower %SS, the U-pT-12 sensor’s ability to function in complex water samples has been verified.

### 3.4. Sensor Response on a Disposable Paper Electrode

Although many electrochemical biosensors were fabricated on conventional gold disk electrodes [40,41,47,48,49,50], there are advantages in employing electrodes that are more cost-effective [39]. Yang et al. developed a simple method to deposit micro- and nano-structured gold on SPCEs and used them in the fabrication of an E-DNA sensor [39]. Gold-plated SPCEs have since been used in the fabrication of several E-AB and metal ion sensors [20,27,55], but they have not been tested extensively with E-PB sensors. Here, we assessed the response of the U-pT-12 sensor that was fabricated on a gold-modified SPCE (Figure S5). The sensor responded to U(VI), but the signal attenuation (72% SS) was slightly lower than that obtained with a gold disk electrode. The difference in probe coverage (~1.8 × 10^12^ molecules cm^−2^) could be responsible for the change in signal attenuation [27,40,41,56]. Furthermore, because of the structure of the electrodeposited gold, it is possible that some of the immobilized peptides were not positioned in a way where binding of U(VI) could occur. This could also affect the total achievable %SS. The sensor was subsequently regenerated using the aforementioned method, and the % signal recovery was close to 100%. Despite the aforementioned differences, we have demonstrated the use of gold-plated SPCEs in the fabrication of an E-PB U(VI) sensor.

### 3.5. Key Analytical Properties of the U-pT-12 Sensor—A Comparison

Table 1 shows the key analytical properties of the U-pT-12 sensor and several previously reported U(VI) sensors and detection methods [9,10,11,12,13,14,15,16,17]. The LOD of the current sensor compares well with several other U(VI) sensors, including colorimetric, SERS, and electrochemical sensors [11,13,14,15]. Although there are sensors with significantly lower LODs [10,12], these sensors require custom designed and synthesized U(VI)-specific ligand and/or nanomaterials. The U-pT-12 sensor only requires purchased peptides and a straightforward 2-step sensor fabrication method. The assay time is slightly shorter when compared to the electrochemical sensor that relies on specific interactions between U(VI) and surface-immobilized DNA [16]. Interactions between U(VI) and the surface-bound probe could be negatively impacted by steric hindrance, even if the probe coverage is considered optimal. Despite the slightly longer assay time, the simple and reagentless design remains the key feature and advantage of the U-pT-12 sensor. With further optimization, it can potentially be used for on-site analysis of U(VI) in environmental samples. 

## 4. Conclusions

We have successfully employed a 12-AA peptide sequence, U-pT-12, as a biorecognition probe for electrochemical detection of U(VI). The peptide sequence originates from the Ca(II) binding site of CaM, but the incorporation of a phosphothreonine has enhanced the peptide’s affinity for U(VI) over Ca(II). Consequently, the U-pT-12 sensor responded optimally to U(VI) and did not cross-react with many of the metal ions tested, including Ca(II), Cd(II), and Cr(VI). The results are in contrast to those obtained with U-12, a peptide probe with a nonphosphorylated threonine, which showed limited response to U(VI). Furthermore, the sensor achieved a LOD well below the EPA MCL for uranium in drinking water. Its ability to function well in two complex water samples was tested and confirmed. This study presents a promising solution for on-site detection of U(VI) in real water samples, in addition to demonstrating the advantages of exploiting metal ion-binding motifs in proteins and incorporation of non-standard AA in the design of metal ion sensors.

## Figures and Tables

**Table 1:** A comparison of the key analytical properties of the U-pT-12 sensor and other U(VI) sensors and detection approaches.

Type of Sensor/Material	Detection Method	LOD (nM)	Dynamic Range or Linear Dynamic Range (µM)	Assay Time (min)	Ref.
Covalent organic framework nanozyme	UV-Vis (Colorimetric)	50	0.18–75	10	[9]
Coordination ligand W1H	UV-Vis (Colorimetric)	9.33	0–16	30	[10]
Hemin-modified metal- organic frameworks	UV-Vis (Colorimetric)	79	0.25–40	3	[11]
Protamine capped gold nanoclusters	Fluorescence	6.1	0.0204–9.74	35	[12]
Citrate-stabilized silver nanoparticles	SERS	60	0.2–5	NR	[13]
Plasmonic nanoparticle	SERS	110	0–13.6	30	[14]
Schiff base ionophore	Electrochemical (Potentiometric)	390	1–100,000	0.15	[15]
DNA-modified electrode	Electrochemical (SWV)	30	0.1–1	60	[16]
Polydopamine/rGO -modified electrode	Electrochemical (DPSV)	50	0.1–50	15	[17]
E-PB U-pT-12 sensor	Electrochemical (ACV)	50	0.05–4.5	~40	This work

Abbreviations: UV-Vis: ultraviolet-visible spectroscopy; SERS: surface-enhanced Raman spectroscopy; NR: not reported; SWV: square wave voltammetry; rGO; reduced graphene oxide; DPSV: differential pulse stripping voltammetry; ACV: alternating current voltammetry.

## Data Availability

The dataset generated and analyzed in this study is not publicly available but may be obtained from the corresponding author upon reasonable request.

## Supplementary Materials

The following supporting information can be downloaded at: https://www.mdpi.com/article/10.3390/bios12110961/s1, Figure S1: Structures of the U-pT-12 and U-12 peptide probes; Figure S2: Schematic illustration of the U-12 sensor and ACVs of the sensor; Figure S3: Scan rate-dependent current profiles for the U-pT-12 sensor; Figure S4: ACVs of the U-pT-12 sensor in the absence and presence of 4, 40, 400, 4000 µM Ca(II), and 4 µM U(VI); Figure S5: ACVs of the U-pT-12 sensor fabricated on a gold-plated SPCE.
