# Supplementary material for: Threonine Phosphorylation of an Electrochemical Peptide-Based Sensor to Achieve Improved Uranyl Ion Binding Affinity
Source: Biosensors (Basel). 2022 Nov 2;12(11):961. doi: 10.3390/bios12110961 (PMC9688285; doi:10.3390/bios12110961)
Supplement: Supplementary file 1 [file biosensors-12-00961-s001.zip › biosensors-1803270-supplementary.pdf]

biosensors

MDPI

Supplementary Material

Threonine Phosphorylation of an Electrochemical Peptide-Based Sensor to Achieve Improved Uranyl Ion Binding Affinity

Channing C. Thompson and Rebecca Y. Lai *

Department of Chemistry, University of Nebraska-Lincoln, Lincoln, NE 68588-0304, USA
* Correspondence: rlai2@unl.edu

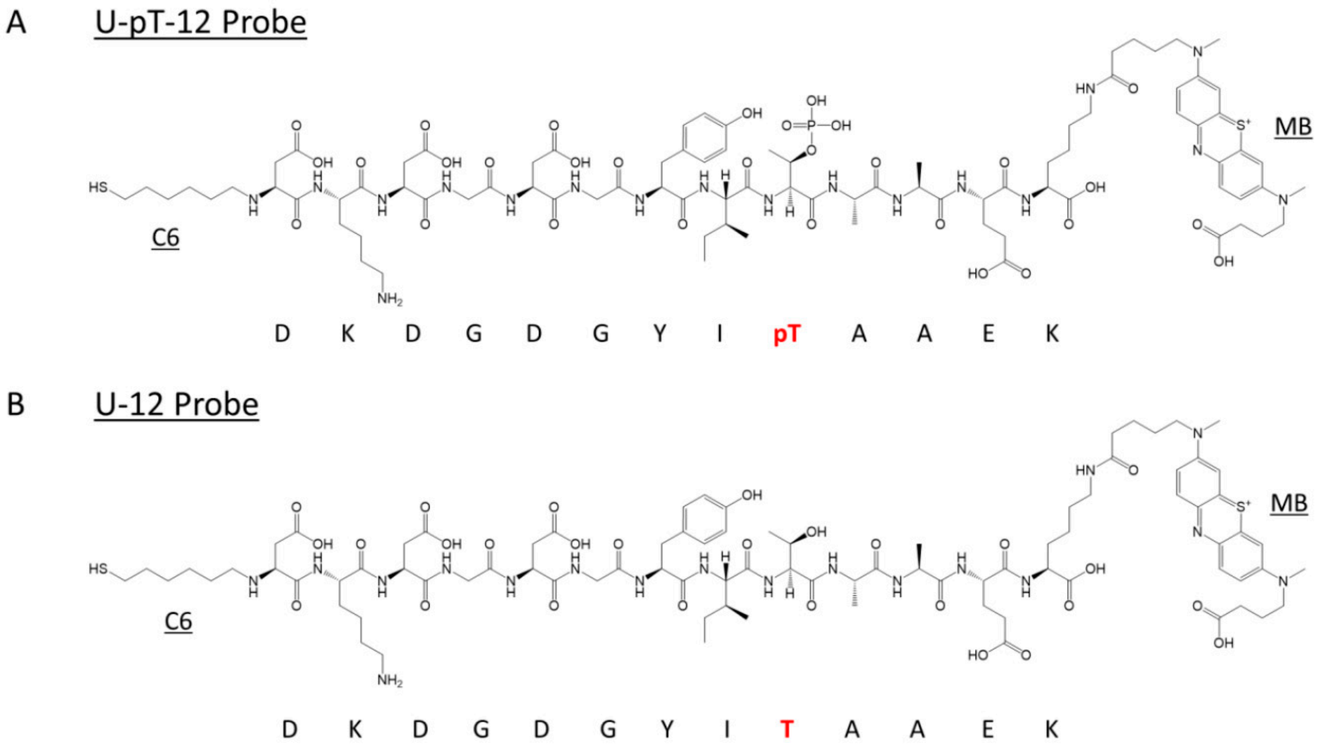

Figure S1. Structures of the (**A**) **U-pT-12** and (**B**) **U-12** peptide probes used in this study.

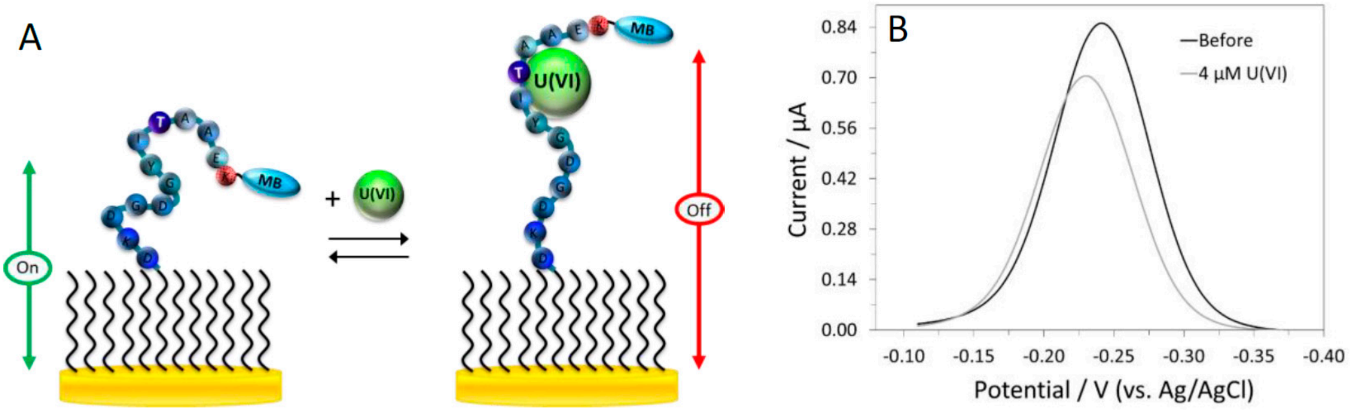

Figure S2. (**A**) Schematic illustration of the E-PB U(VI) sensor fabricated using the **U-12** peptide probe. (**B**) CVs of the sensor recorded at 10 Hz in a Phys2 buffer in the absence and presence of 4 µM U(VI).

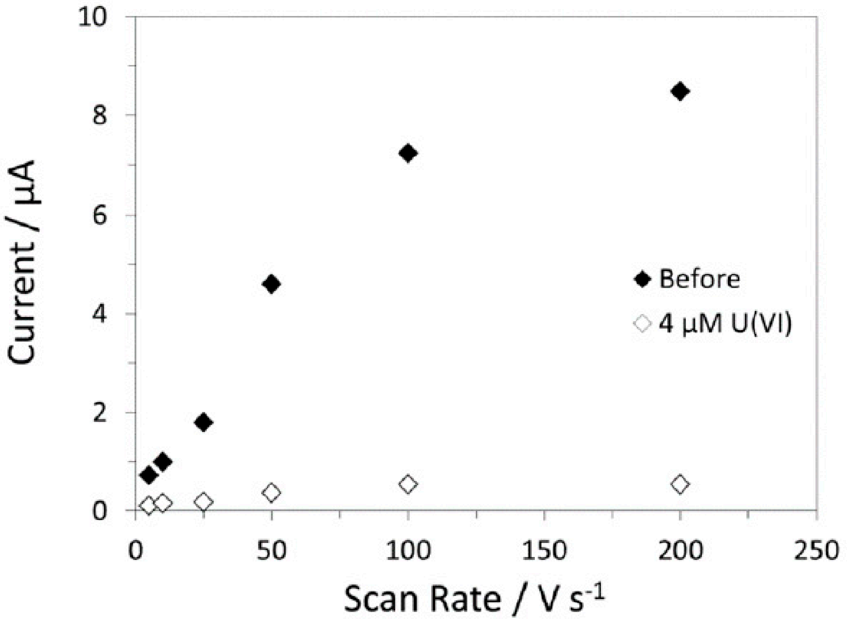

Figure S3. CV scan rate-dependent current responses of the **U-pT-12** sensor in the absence and presence of 4 µM U(VI) in a Phys2 buffer. The cathodic peak currents were used to obtain this plot; however, the anodic peak currents could also be used, and the results were found to be similar.

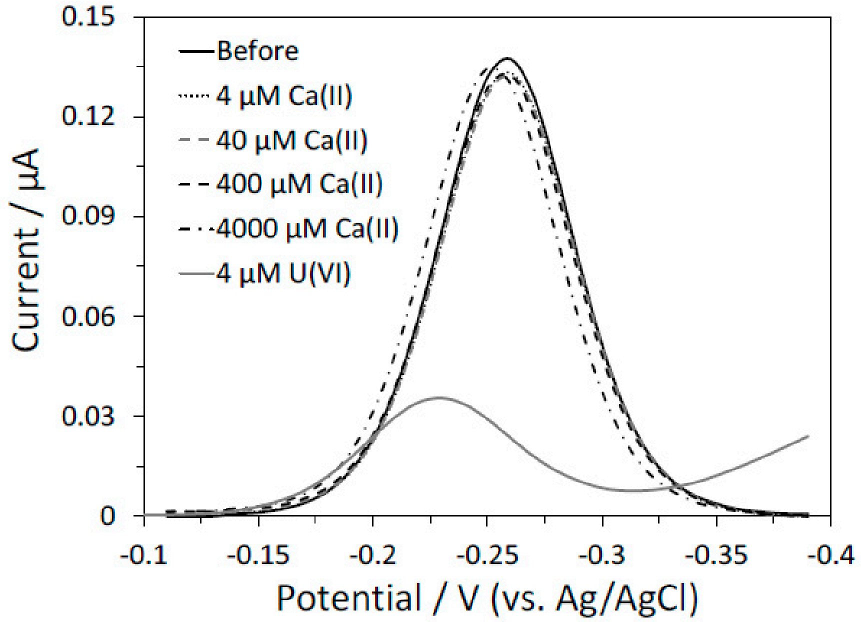

Figure S4. ACVs of the **U-pT-12** sensor recorded at 10 Hz in a Phys2 buffer in the absence and presence of 4, 40, 400, 4000 µM Ca(II), and 4 µM U(VI).

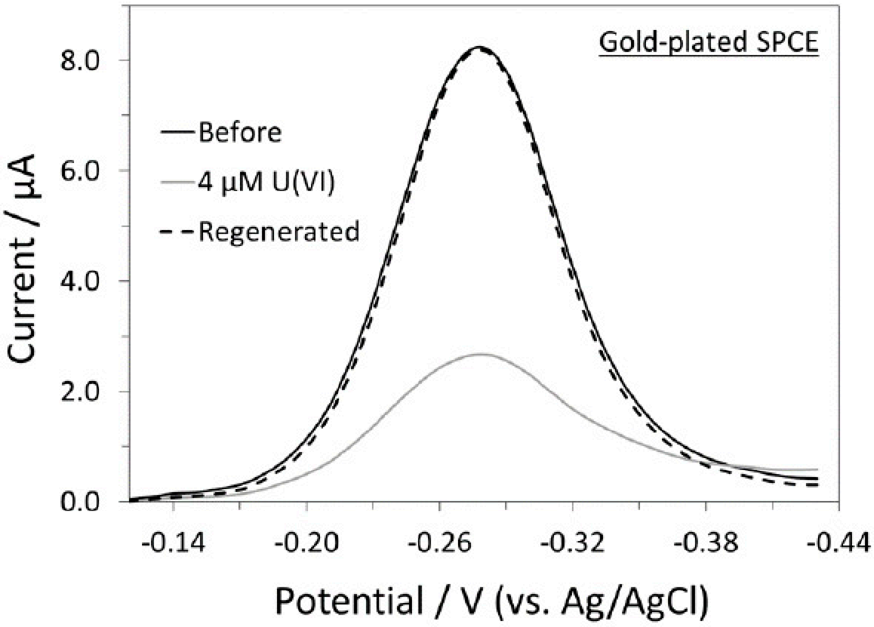

Figure S5. ACVs of the **U-pT-12** sensor fabricated on a gold-plated SPCE in the absence and presence of 4 μM U(VI), and after sensor regeneration.
